# Complications of Minimally Invasive, Tubular Access Surgery for Cervical, Thoracic, and Lumbar Surgery

**DOI:** 10.1155/2014/451637

**Published:** 2014-07-07

**Authors:** Donald A. Ross

**Affiliations:** ^1^Section of Neurological Surgery, Operative Care Division, Portland Veterans Administration Hospital, Portland, OR, USA; ^2^Department of Neurological Surgery, Oregon Health & Science University, 3303 SW Bond Avenue, CH8N, Portland, OR 97239, USA

## Abstract

The object of the study was to review the author's large series of minimally invasive spine surgeries for complication rates. The author reviewed a personal operative database for minimally access spine surgeries done through nonexpandable tubular retractors for extradural, nonfusion procedures. Consecutive cases (*n* = 1231) were reviewed for complications. There were no wound infections. Durotomy occurred in 33 cases (2.7% overall or 3.4% of lumbar cases). There were no external or symptomatic internal cerebrospinal fluid leaks or pseudomeningoceles requiring additional treatment. The only motor injuries were 3 C5 root palsies, 2 of which resolved. Minimally invasive spine surgery performed through tubular retractors can result in a low wound infection rate when compared to open surgery. Durotomy is no more common than open procedures and does not often result in the need for secondary procedures. New neurologic deficits are uncommon, with most observed at the C5 root. Minimally invasive spine surgery, even without benefits such as less pain or shorter hospital stays, can result in considerably lower complication rates than open surgery.

## 1. Introduction

Minimal access spinal surgery is a rapidly developing set of techniques, which have compared favorably with open surgeries in the recent literature (see review in Wong et al., 2012) [1–4]. In addition to reduced blood loss, shorter operative time, reduced postoperative pain, earlier discharge, rapid return to normal activities, and other reported advantages of minimally invasive surgery, [5] a decreased complication rate associated with these surgeries has also been noted, particularly with respect to wound infections [6]. The author reports experience on management of a large series of minimally invasive spine procedures.

## 2. Methods

### 2.1. Patient Population

The author began using the Metrx Tubular Retraction System (Medtronic, Minneapolis) in 2001. This report constitutes a retrospective review of all consecutive spine cases done using this system from that time to the present. Information was obtained from the author's personal surgeries database. This report does not include intentionally intradural procedures or fusion procedures. This series does not include the use of expandable tubes or other minimal access retractor systems other than a tubular system. Procedures reported here were for laminectomy and/or foraminotomy for spondylotic diseases such as discectomy or stenosis, for epidural masses such as metastases, abscesses, or synovial cysts, or for spinal cord stimulator paddle electrode implantation.

### 2.2. Surgical Approach

In each procedure, the patient was positioned prone on a Jackson Table (Mizuho OSI, Union City, CA). Care was taken not to hyperextend the cervical spine. Neuromuscular blockade was not used after induction of anesthesia. Nerve root/spinal cord monitoring was not routinely used. A single dose of preoperative antibiotic was used, usually weight adjusted cefazolin or vancomycin in the case of contraindication to cephalosporins. Ioban (3M, St. Paul, MN) drapes were used in all cases except in cases of iodine allergy. Placement of the tubular retractor system was done according to standard procedure, using first anterior-posterior fluoroscopy for initial placement followed by lateral fluoroscopy for final positioning of the tube. The tubes were affixed to the table mount. For tubes of up to 6 cm in length, an 18 mm diameter tube was used. As the depth increased beyond 6 cm, 20 or 22 mm tubes were used to increase the degree of freedom at depth.

A paramedian approach was used for lumbar laminectomy or discectomy and for cervical laminectomy or foraminotomy. A far lateral approach was used for lumbar foraminotomy or far lateral discectomy, with the incision lateral to the pars interarticularis. Bilateral canal decompression was performed by angling the tube toward the contralateral side after removal of some of the base of the spinous process. For spondylotic stenosis, attention was paid to directing the tube to the site of maximal canal stenosis, usually just below the disc space where the ligamentum flavum is most compressive of the thecal sac.

The microscope was used for visualization throughout the procedure. No monopolar coagulation was ever used during these procedures. A 5 mm diamond drill under continuous automatic irrigation was used for bone removal. Hemostasis was obtained with low power bipolar coagulation, bone wax, and NuKnit (Ethicon). Copious irrigation was done with lactated ringers without antibiotic. Multilevel cervical procedures were done by wanding the tube to redirect it to another level without removing it. Two-level lumbar procedures were performed by removing the tube and redirecting it through the same incision to the additional levels. Three-level lumbar procedures were done through two incisions, using one incision for two levels and a second incision for the third. In these cases, an 8-French-red-rubber tube was left in at the initial level as a suction drain to prevent epidural hematoma accumulation and then removed at the end of the procedure. Rarely, a 3/32 Hemovac (Zimmer, Warsaw, IN) drain was left in place for a few hours postoperatively if hemostasis was not perfect. Fluoroscopic verification of the rostrocaudal extent of decompression was always obtained using a nerve hook and a ball hook to define the points of maximal rostral and caudal canal decompression.

When a durotomy occurred, if possible, suture repair was performed using a 4-0 silk suture on a very small round needle using the micropituitary forceps from the Metrx set as the needle holder. Instrumentation is now available to facilitate dural closure (Haque et al., 2013; [7] Scanlan International, St. Paul). In some cases, a small piece of NuKnit (Ethicon) or dural substitute was placed intradurally such that it was pushed flat up against the internal side of the dura by the arachnoid to prevent nerve root herniation through the durotomy. Fibrin glue (Tisseel, Baxter) was placed over the durotomy prior to closure. The wound closure was not altered from that described below. Lumbar patients were kept in 3 degrees of Trendelenburg for 6–12 hours postoperatively and then mobilized if a 2-hour test period of sitting up 30 degrees did not produce a postural headache.

After obtaining hemostasis at the dural level, the retractor tube was slowly withdrawn under the microscope, and hemostasis was assured in the muscle layers. The lumbodorsal, thoracodorsal, or cervical fascia and Scarpa's layer were independently closed with 2-0 absorbable suture on a curved urology needle. In some obese patients, the fascia could not be reached for closure and only Scarpa's layer could be closed. The dermis was closed with inverted 3-0 absorbable suture. No skin suture was used. Mastisol and a single longitudinal Steri-strip (3 M) were used for the skin. A simple band-aid was placed. Patients were allowed to shower 48 hours postoperatively. Patients were instructed that a bump would be visible at the incision for several months before resolving. All patients were seen postoperatively at 4 weeks and 3 months after the procedure. If the patient was doing well, no further follow-up was performed after that time. Patients with ongoing problems were followed until a therapeutic endpoint was reached. The author has been in practice in the State of Oregon for 15 years and patients in need of further care have been able to contact him continuously throughout that time.

## 3. Results

In the 12-year period from 2001 to 2013, 1231 cases were performed using Metrx tubes, excluding fusion procedures and intentionally intradural procedures such as intradural tumors, vascular malformations, or hematomas. Case distribution is detailed in Table 1: 262 cases were cervical, 40 cases were thoracic, and 929 cases were lumbar. Patient age ranged from 16 to 95 years (mean 53 ± 18 years; standard deviation). No patient was excluded from this type of surgery based upon weight.

There were no direct nerve root or spinal cord injuries or new motor neurologic deficits after surgery with the exception of the C5 root. Three patients of a total of 53 foraminotomy patients operated at the C5 level (5.7%) developed delayed C5 weakness within a few days of surgery. Two patients made complete recoveries within three months and one never regained function of the C5 root. For comparison, during the author's career, 46 patients had an anterior cervical surgery including the C5 root level for radiculopathy and 76 patients for myelopathy. One of these 122 patients had a transient C5 palsy (0.8%). Thus, for this entire series of 1231 procedures, there were 3 motor deficits (0.24%) and one permanent motor deficit (0.08%). Two patients noted increased sensory deficits in the dermatomal distribution of the operated cervical nerve root (one C6 and one C8), both of which resolved over 2 months. One patient early in the series developed a symptomatic epidural hematoma at the first level of a two-level lumbar laminectomy which required evacuation the night of surgery. There were no neurologic sequelae of this event, but the author began using the red rubber drain as noted above after this event. Another patient early in the series developed pain after a durotomy resulted in a nerve root herniation requiring reexploration from the contralateral side to reduce this. The use of NuKnit or artificial dura to prevent nerve root herniation has prevented this from happening again (see above). Overall, there were 33 durotomies in 1231 patients (2.7%).

There were 295 first time lumbar discectomies, one two-level lumbar discectomy, and 55 redo discectomies. There were 88 procedures for lumbar far lateral discectomies and 11 for redo far lateral discectomies. 186 procedures were performed for lumbar spinal stenosis at a single level, 59 for two-level decompressions (one of which was done at nonadjacent levels), and 2 for three-level decompressions. Forty-four lumbar procedures were for resection of synovial cysts. Lumbar epidural abscesses were drained in 15 cases. Two procedures were for lumbar epidural metastatic tumor resections and one was for a spontaneous lumbar epidural hematoma. 149 procedures were for single-level lumbar foraminotomy, 21 were for two-level lumbar foraminotomy, and one was for bilateral foraminotomy at the same level.

In total, there were 929 lumbar procedures. There were no infections in these patients during the 3-month postoperative follow-up and no delayed infections that have been brought to the author's attention. There were no neurologic injuries in these patients. A single patient early in the series undergoing a two-level lumbar laminectomy for stenosis developed a symptomatic epidural hematoma at the first level several hours after surgery requiring evacuation with no permanent sequelae. There have been no further events since the use of drains during the second level procedure as described above. There were 12 durotomies in the 295 (4.1%) first time lumbar discectomies, 3 durotomies in the 55 (5.5%) redo lumbar discectomies, 7 durotomies in the 186 (3.8%) single-level lumbar laminectomies for stenosis, 6 durotomies in the 59 (10.1%) multilevel lumbar laminectomies, 1 durotomy in the 44 (2.3%) synovial cyst resections, 2 durotomies in the 171 (1.2%) lumbar foraminotomies, and one durotomy in the 99 (1%) far lateral discectomies. Thus, over the entire 929 patients, there were 32 durotomies (3.4%). No patient developed postural headaches, CSF leaks, symptomatic pseudomeningocele, or requirement for any additional intervention for CSF leak in the 3-month minimum period of follow-up after surgery.

There were 160 first time single-level cervical foraminotomies. There were 72 two-level cervical foraminotomies, 9 redo cervical foraminotomies, and one bilateral cervical foraminotomy. Six cervical laminectomies were performed for canal stenosis, one for epidural tumor, one for synovial cyst, and one for an acute epidural hematoma.

In total, there were 262 cervical procedures. There were no infections in these patients. Two patients noted transient increased hypesthesia in the dermatomal distribution of an operated nerve root after surgery, but these both resolved within 2 months. There were no motor deficits other than C5 as noted above. There were no durotomies in these patients.

There were 7 thoracic discectomies, 6 thoracic laminectomies for stenosis, 4 thoracic biopsies, 3 thoracic epidural tumor resections, and one drainage procedure for a thoracic epidural abscess. Twenty spinal cord stimulator paddle electrodes were implanted. In total, there were 40 thoracic procedures. There were no wound infections, new neurologic deficits, and one durotomy. One paraplegic patient developed fatal staphylococcal sepsis 6 weeks after surgery, presumed to be due to one of many decubitus ulcers, with no evidence of wound infection at the time of his demise.

## 4. Discussion

While there is accumulating data supporting minimally invasive spine surgery techniques, recent reviews still suggest the need for more Level I and II data to demonstrate benefit over open surgery [3]. Reduced complication rates as a benefit of tubular retractor based surgeries may factor into the decision of when to use these techniques.

There were no wound infections in this series of 1231 cases. Without any infections, it was not possible to assess the role of diabetes, obesity, timing of perioperative antibiotics, or anterior versus posterior approaches in infection, but these have been reported to be risk factors by others [8, 9]. Wound infection rates after open spine surgery have been reported to be in the range of 0.7 to 16% [6]. In O'Toole et al.'s review [6] of 1338 cases performed by three surgeons through tubular retractors, which included fusions and intradural procedures, there were 2 superficial wound infections (both in fusion procedures) and one deep infection, discitis in a decompressive laminectomy in a 90-year-old patient. This patient had suffered from streptococcal cellulitis and urinary tract infection 2 months postoperatively and grew the same organism from his disc space at needle biopsy, presumably by hematogenous spread and not by direct contamination at surgery. The infection rate for decompression only was 0.1%, for fusion 0.74%, and overall 0.22%. They concluded that minimally invasive techniques may reduce postoperative wound infection by as much as 10-fold compared with modern series of open procedures. Ikuta et al. [10] reported on 114 consecutive patients undergoing minimally invasive lumbar decompression for stenosis. There were no surgical site infections noted. Summing these series with the present data, there were 1039 plus 114 plus 1231 = 2384 noninstrumented, extradural procedures with only a single deep wound infection which is likely to have been due to secondary hematogenous seeding from an unrelated source.

O'Toole et al. [6] speculate on four potential reasons for the low surgical site infection rate: (1) reduced tissue exposure as much of the wound is covered by the tubular retractor, (2) minimal exposure of the skin to the wound, with little opportunity for instruments or the surgeons hands to contact the skin, (3) smaller wounds which are more likely to heal rapidly, and (4) reduction in dead space with resulting hematoma and seromas which can serve as the nidus for infection.

The author agrees and adds (5) lack of use of monopolar coagulation which minimizes devitalized tissue in the wound, another potential source of infection, (6) symmetric distribution of retraction forces which minimizes the risk of tissue ischemia and necrosis, (7) the incision which is made in a single stroke, as the number of knife passes has been shown to increase the infectability of experimental wounds, [11] and (8) the absence of skin sutures, which may allow an avenue of access to the wound.

Postoperative C5 root palsy is an enigmatic entity. It has been reported to occur in 5.1% of all cervical cases including the C5 level and 8.7% of posterior decompressions at the C5 level [12]. Another study reported an incidence of 12% in 98 patients undergoing either anterior or posterior approaches without foraminotomy [13]. This study found that the likelihood of a postoperative palsy was higher in patients with narrower spinal canal anterior posterior diameter, with narrower foraminal diameter, and with increases in the cord lamina angle at the operated level [13]. The use of cooled saline during drilling has been reported to reduce the incidence of new neurologic deficits after cervical laminoplasty, [14] but if heating related to drilling was the cause of root palsy, then C5 would not be the only affected root. Therefore, there remains something unique to the C5 root's vulnerability [13].

New neurologic deficit after elective spinal surgery has been reported based upon the Scoliosis Research Society database [15]. Of 108,419 cases, new neurologic deficits were documented for 1064 (1.0%), including 662 nerve root injuries, 74 cauda equine injuries, and 293 spinal cord injuries (deficit type was not specified for 35 cases). The rate of new neurologic deficits for cases with implants was more than twice that for cases without implants (1.15% versus 0.52%). Therefore, the lower rate of 0.52% is more comparable with the current series and indicates that minimally invasive surgery can be performed with acceptable neurologic complication rates when compared to such a large database.

Durotomy is a common complication of spinal surgery. In 1014 procedures for 1261 levels of lumbar spinal stenosis, Takahashi et al. [16] reported durotomy in 4% of cases and 3.3% of operated levels. Durotomy was highest with juxtafacet cysts (18.2%) and spondylolisthesis (9%). In the SPORT analysis, [17] 9% of 409 patients undergoing first time open laminectomy had a durotomy. Durotomy prolonged surgery time, blood loss, and inpatient stay, but there was no difference in primary outcomes at follow-up [17]. In the United Kingdom, prospective data on 1549 cases showed an incidence of durotomy to be 3.5% for primary discectomy, 8.5% for spinal stenosis surgery, and 13.2% for revision discectomy [18]. Durotomy appears to be less common during tubular retraction based surgery than that reported in these open surgical series and the consequences of such appear to be reduced. With no wound cavity in which to accumulate a pseudomeningocele and no easy route of egress from the wound, delayed complications of CSF leakage appear to be reduced by tubular retraction surgery.

Minimally access spinal surgery using tubular retractors, in properly selected cases, does appear to result in a reduced rate of wound infection, durotomy, symptomatic CSF leak, and new neurologic deficit. In turn, this may result in reduced costs of health care.

## 5. Conclusion

In the author's experience, minimally invasive spine surgery undertaken through tubular retractors shows a low wound infection rate when compared to open surgery. Durotomy is no more common than open procedures and does not result in the need for secondary procedures. New neurologic deficits are uncommon; however, when present, they are most common at the C5 root.

## Study Limitations

This study was undertaken in an effort to describe the author's personal experience. The retrospective review exposes the study to inherent observational bias. The author has however made every effort to provide an accurate account of the data available.

## Figures and Tables

**Table 1:** Surgical case distribution.

Surgery	Procedure	Number (*n*)	Durotomy (*n*)
Lumbar	Paramedian discectomy	295	12
	Redo discectomy	55	3
	Two-level discectomy	1	0
	Far lateral discectomy	88	1
	Redo far lateral discectomy	11	0
	Stenosis, single level	186	7
	Stenosis, multilevel	59	6
	Stenosis, redo	1	0
	Synovial cyst	44	1
	Epidural abscess	15	0
	Other	3	0
	Foraminotomy, single level	149	1
	Foraminotomy, multilevel	21	1
	Foraminotomy, bilateral	1	0
	Total lumbar	**929**	**32**

Cervical	Foraminotomy, single level	160	0
	Foraminotomy, two-level	72	0
	Foraminotomy, bilateral	1	0
	Foraminotomy, discectomy	11	0
	Foraminotomy, redo	9	0
	Laminectomy for stenosis	6	0
	Laminectomy for tumor	1	0
	Laminectomy for epidural hematoma	1	0
	Laminectomy for synovial cyst	1	0
	Total cervical	**262**	**0**

Thoracic	Discectomy	7	1
	Laminectomy for stenosis	5	0
	Laminectomy for biopsy	4	0
	Laminectomy for abscess	1	0
	Laminectomy for tumor	3	0
	Laminotomy for electrode	20	0
	Total thoracic	**40**	**1**
